# Antimicrobial resistance in food-producing animals: towards implementing a one health based national action plan in Israel

**DOI:** 10.1186/s13584-023-00562-z

**Published:** 2023-04-26

**Authors:** Tali Sarah Berman, Zohar Barnett-Itzhaki, Tamar Berman, Eli Marom

**Affiliations:** 1 Mimshak, The Israel Society of Ecology and Environmental Sciences, 19 Kehilat New York St, Tel Aviv, Israel; 2 Ruppin Research Group in Environmental and Social Sustainability, Ruppin Academic Center, 4025000 Emek Hefer, Israel; 3 Public Health Services, Ministry of Health, 39 Yirmiyahu St, Jerusalem, Israel; 4 Present Address: Department of Entomology, Newe Ya’ar Research Center, ARO, Ramat Yishai, Israel

**Keywords:** Antibiotics, Antimicrobial resistance surveillance systems, Growth promotors, Health policy, One Health, Veterinary medicine

## Abstract

**Background:**

Development of antimicrobial resistance poses a major threat to human and animal health worldwide. Antimicrobials are frequently used in animal husbandry, making food-producing animals a widespread and important source of antimicrobial resistance. Indeed, recent evidence demonstrates that antimicrobial resistance in food-producing animals poses a threat to the health of humans, animals and the environment. To address this threat, national action plans have been implemented based on a ‘One Health’ approach, which integrates actions across human and animal health sectors to combat antimicrobial resistance. Although under development, Israel has yet to publish a national action plan against antimicrobial resistance, despite alarming findings of resistant bacteria in food-producing animals in the country. Here we review several national action plans against antimicrobial resistance around the world in order to suggest approaches to develop a national action plan in Israel.

**Main body:**

We investigated worldwide national action plans against antimicrobial resistance based on a ‘One Health’ approach. We also conducted interviews with representatives of relevant Israeli ministries to understand antimicrobial resistance policy and regulatory frameworks in Israel. Finally, we present recommendations for Israel towards implementing a ‘One Health’ national action plan against antimicrobial resistance. Many countries have developed such plans, however, only a few are currently funded. Furthermore, many countries, especially in Europe, have taken action to reduce the use of antimicrobials and the spread of antimicrobial resistance in food-producing animals by banning the use of antimicrobials to promote growth, reporting data on the use and sales of antimicrobials in food-producing animals, operating centralized antimicrobial resistance surveillance systems and preventing the use of antimicrobials important to human medicine to treat food-producing animals.

**Conclusions:**

Without a comprehensive and funded national action plan, the risks of antimicrobial resistance to the public health in Israel will escalate. Thus, several actions should be considered: (1) Reporting data on the use of antimicrobials in humans and animals. (2) Operating a centralized surveillance system for antimicrobial resistance in humans, animals and the environment. (3) Improving awareness regarding antimicrobial resistance in the general public and in health practitioners from both human and animal sectors. (4) Composing a list of critically important antimicrobials to human medicine that’s use should be avoided in food-producing animals. (5) Enforcing best practices of antimicrobial use at the farm-level. (6) Reducing incidence of infection through farm biosecurity. (7) Supporting research and development of new antimicrobial treatments, vaccines and diagnostic tools.

## Background

Since their discovery in the twentieth century, antibiotics have had an immense impact on human and animal health by enabling the treatment of infectious diseases. Nowadays, industrial agriculture relies heavily on the use of antimicrobials (*e.g*. antibiotics, antifungals and antiprotozoals) to treat and prevent disease, improve animal welfare and increase productivity [1]. However, large scale use of antimicrobials may result in the development of resistance in bacteria [2].

Antimicrobial resistance is the ability of microorganisms to become resistant to antimicrobials which they were previously susceptible to, reducing treatment effectiveness [2]. Antimicrobial resistance can be acquired by genetic mutations, but it is mainly acquired through horizontal gene transfer (HGT), *i.e.*, the acquisition of new genetic elements, via transduction (genetic elements transferred by bacteriophages), transformation (direct uptake and incorporation of genetic elements from the environment) or conjugation (transfer of genetic elements between bacterial cells). Although the development of antimicrobial resistance is a natural phenomenon, extensive and inappropriate use of antimicrobials can greatly enhance it, leading to a rapid spread of resistant bacterial communities [2, 3].

Indeed, antimicrobial resistance is one of the major public health concerns of the twenty-first century, with significant economic implications [2]: according to the World Health Organization, infections caused by antibiotic-resistant bacteria are accountable for at least 700,000 annual deaths worldwide, a number that could rise to 10 million by 2050 if no substantial action is taken [4]. According to the Lancet’s latest report, this number is much higher—in 2019 alone, nearly five million deaths were estimated to be associated with antimicrobial resistance [5]. Antibiotic‐resistant infections are expected to cost the global economy 100 trillion USD by 2050 [6].

The use of antimicrobials in food-producing animals has increased in the past decades due to a rise in the global demand for animal protein [2] and due to cultural changes [7]. The majority of antimicrobials sold across the world (73%) are intended for animals raised for food [8]. These antimicrobials are not only used to treat infectious diseases, but they are also used in sub-therapeutic levels for growth promotion and for prevention of diseases (*i.e*., prophylactic use—administration of antimicrobials to animals at high risk of disease; metaphylactic use—treatment of a group of animals, which are in close contact, without evidence of disease). Over the past years, it has become evident that the extensive use of antimicrobials in food-producing animals has contributed to the spread of antimicrobial resistance and thus reduced available treatment options. Examples include Carbapenem resistant bacteria in poultry, pigs and cattle [9–11]; multi resistant *Escherichia coli* in poultry [12] and Cefotaxime resistant bacteria in cattle [13].

While extensive use of antimicrobials in animal husbandry constitutes an important source of antimicrobial resistance, it is not entirely clear how this phenomenon affects human health. Although it is difficult to establish the direction of movement of resistance between humans and food-producing animals [14], evidence from recent years suggests that antimicrobial resistance in food-producing animals poses a threat to human health. For example, the European Centre for Disease Prevention and Control (ECDC), the European Food Safety Authority (EFSA) and the European Medicines Agency (EMA) produced joint reports in 2015 and 2017, which established significant links between the use of Fluoroquinolones in food-producing animals and resistance in indicator *E. coli*, *Salmonella spp., Campylobacter jejuni* and *Campylobacter Coli* in animals and humans. Significant links were also observed between the use of Tetracycline in food-producing animals and resistance in indicator *E. coli, Salmonella spp.* and *C. Jejuni* [15, 16]*.* This is especially problematic when the antimicrobials used in food-producing animals are closely related to, or are the same as antimicrobials prescribed to humans. For example, Colistin, a last resort antibiotic for the treatment of multi‐drug resistant bacteria in humans, is used as a feed additive for animals around the world. In 2015, the first mobile mechanism of Colistin resistance (MCR-1) was reported in humans and food-producing animals in China [17]. Since then, MCR-1 has become widespread around the world [18].

Antimicrobial resistance and antibiotic-resistant bacteria can be transmitted directly from food-producing animals to humans through direct contact with animals and through consumption of animal products, especially when handled or cooked inadequately [14, 19]. According to the United States Centers for Disease Control and Prevention (CDC), about 1 in 5 resistant infections are caused by bacteria from food and animals [20]. Indirect transmission to humans can occur through contaminated crops and water supplies [14, 19]. Moreover, antimicrobial resistance can potentially spread into the environment through animal waste. For example, the presence of resistant bacteria in food-producing animal wastewater, facilitates the transfer of antibiotic resistant genes (ARGs) in wastewater treatment plants [21]. These resistant genes accumulate in the sludge, creating environmental reservoirs of antimicrobial resistance which in time may be transmitted to human (and animal) pathogens or microbiota [3, 14, 21, 22]. Since the spread of antimicrobial resistance is linked between humans, animals and the environment, a multisectoral approach which integrates actions across all health sectors is required to effectively address antimicrobial resistance (*i.e.*, the “One Health” approach; [23]).

Studies conducted in recent years show that antimicrobial resistance is also widespread among food-producing animals in Israel. A national survey of cattle farms revealed high prevalence of ESBLPE (extended spectrum beta lactamase producing Enterobacteriacea; [24, 25]). Another study demonstrated transmission of a Methicillin-resistant *Staphylococcus aureus* between horses and humans [24]. A large national survey of 13 common poultry‐associated *Salmonella* revealed that 60% of tested isolates were multidrug resistant [26]. Furthermore, the emergence of a multidrug resistant *Salmonella Muenchen* strain, that harbors an epidemic megaplasmid (pESI) which can be self-mobilized into *E. coli* and other *Salmonella* serovars, has been recently identified in Israel [27]. In the aquaculture sector, fish ponds treated with antimicrobials harbored resistant *Aeromonas* strains [28]. High *E. coli* resistance to Ampicillin and Sulphonamides have been demonstrated in all animal sectors [29]. According to the 2020 annual report published by the Central Laboratories in the Ministry of Health of Israel, *E. coli O157:H7 serotype, Salmonella spp. and Campylobacter spp.* (bacteria acquired from animals) demonstrate high multi-drug resistance rates [30]. Despite these alarming findings, Israel has yet to implement a national action plan against antimicrobial resistance in humans and animals.

In this paper, we review national action plans against antimicrobial resistance that were established in different countries, with a focus on plans based on the ‘One Health’ approach [23]. We also conducted interviews with representatives of the Israeli Ministry of Health and the Israeli Ministry of Agriculture and Rural Development to obtain information regarding antimicrobial resistance policies and relevant regulatory frameworks in Israel. We then highlight key recommendations for Israel regarding prudent use of antimicrobials in food-producing animals, towards implementing a ‘One Health’ national action plan against antimicrobial resistance.

## Methods

We searched Google Scholar and official government websites for documents in English which were published between 2010 and 2022. The keywords included: “antimicrobials”, “antimicrobial resistance”, “antibiotics”, “antibiotic resistance”, “food-producing animals”, “livestock”, “poultry”, “animals”, “agriculture,” “national action plan” and “one health”. We then examined national action plans and policies related to antimicrobial resistance in selected developed countries: the European Union, the United States of America, Australia, and Japan (see Table 1); representative Middle Eastern countries: Egypt, Jordan and Lebanon, and compared them to Israel. Additional information was obtained from the World Health Organization website.

**Table 1:** Summary of policies and actions against antimicrobial resistance in the food-producing animal sector in Israel and selected developed countries

	Israel	European Union countries	United states of America	Australia	Japan
Use of growth promoters	Prohibited since 2018	Prohibited since 2006	Prohibited since 2017	Restrictions only on the use of antimicrobials important to human health	Partially restricted—Colistin is prohibited
Collecting data on sales and use of antimicrobials	None, except for a voluntary survey on sale data form wholesalers conducted in 2014 by the Veterinary Services and Animal Health unit of Israel	Started voluntarily in 2009 and became obligatory in 2019	Data regarding antimicrobial sales by animal species collected since 2016	Data regarding antimicrobial sales in the country are partial (last report from 2014)	Data regarding antimicrobial sales by animal species collected since 2000
Monitoring antimicrobial resistance in food-producing animals	No centralized system—antimicrobial resistance in foodborne pathogens is monitored by the Government Central Laboratories in MOH. Antimicrobial resistance in pathogens of animals is partially monitored by the Kimron Veterinary Institute in the Ministry of Agriculture and Rural Development	Not yet coordinated at the European Union level, however many countries monitor antimicrobial resistance in food-producing animals	Data regarding antimicrobial resistance in food-producing animals, retail meat products, and foodborne pathogens are collected by the US Food and Drug Administration, the US Department of Agriculture and the US Centers for Disease Control and Prevention as part of the National Antimicrobial Resistance Monitoring System	None	Monitored by the Japanese Veterinary Antimicrobial Resistance Monitoring System
List of critically important antimicrobials to human medicine	None. Partially adopted the European Union list (category A)	European Union list	The US Food and Drug Administration published a list in 2003, which has yet to be revised since	Regional adapted list published in 2018	Regional adapted list published in 2014
Current national One Health action plan against antimicrobial resistance	Yet to be implemented	‘A European One Health action plan against antimicrobial resistance, published in 2017	‘National action plan for combating antimicrobial resistance 2020-2025’, published in 2020	‘Australia’s National Antimicrobial Resistance Strategy—2020 and beyond’, published in 2020	‘National Action Plan on antimicrobial resistance 2016-2020’, published in 2016

**Fig. 1:**
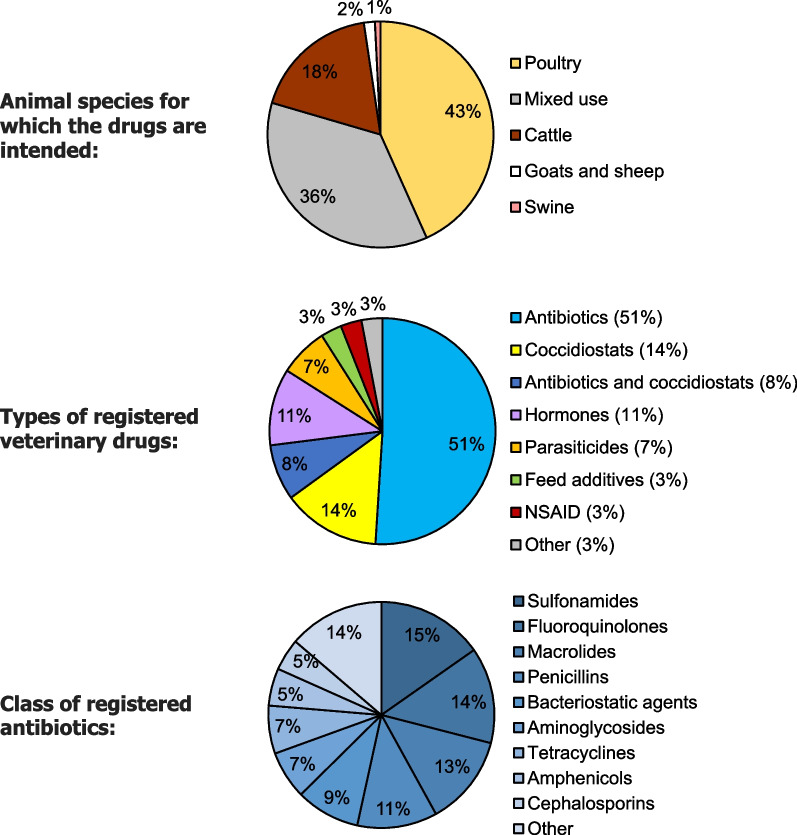
Veterinary drugs registered for food-producing animals in Israel 2020. Data was obtained from the Pharmaceutical Administration in the Ministry of Health

We conducted face-to-face interviews with representatives of the Israeli Ministry of Health and with representatives of the Israeli Ministry of Agriculture and Rural Development to obtain information regarding: (1) The veterinary drug regulatory system in Israel. (2) Actions taken by the Israeli government to combat antimicrobial resistance in the food-producing animal sector. (3) The development and implement of a national action plan against antimicrobial resistance in Israel. We obtained data on veterinary drugs registered for food-producing animals in Israel and on non-registered veterinary drugs imported to Israel from the Pharmaceutical Administration in the Ministry of Health (used for Fig. 1 and Table 2, see results section).

**Table 2:** Non-registered veterinary drugs imported to Israel between 2017–2019 under article 29 C of the pharmacist regulation

	Imported unregistered veterinary drugs			Registered veterinary drugs
	2017	2018	2019	
Number of drugs	380	393	388	349
Number of drugs intended for food-producing animals	113	101	144	215
Number of active substances	46	45	55	–
Percent of antimicrobial substances	56%	44%	76%	59%
Most common antimicrobial substances	Cephalosporin	Cephalosporin	Penicillin	Sulfonamides, Fluoroquinolones and Macrolides

The right column represents data regarding registered veterinary drugs. Data was obtained from the Pharmaceutical Administration in the Ministry of Health

## Results

### Combating antimicrobial resistance: world-wide policies and action plans

### Global

#### Action plans against antimicrobial resistance

Worldwide efforts to address antimicrobial resistance have progressed over the last two decades, leading up to the adoption of the World Health Organization’s Global Action Plan on antimicrobial resistance in 2015 [31]. This plan, based on the ‘One Health’ approach, included five strategic objectives meant to address the threat of antimicrobial resistance by: (1) improving awareness and understanding of antimicrobial resistance, (2) improving knowledge through surveillance, (3) reducing incidence of infection, (4) optimizing the use of antimicrobial agents, and (5) ensuring sustainable investment in countering antimicrobial resistance [32]. This plan called for all countries to develop national action plans by 2017. According to the 2021 World Health Organization tripartite antimicrobial resistance country self-assessment survey (TrACSS), meant to monitor the implementation of national action plans around the world: of the 70% of member states that responded to the survey (136/194), 88% reported having a developed national action plan, but only 20% reported funding their national action plans [33]. The Israeli government’s response to this survey showed that it has yet to develop or implement a national action plan against antimicrobial resistance.

#### Policy and actions in the food-producing animal sector

The World Health Organization published the first list of critically important antimicrobials for human medicine in 2005 [34]. This list ranked antimicrobials according to their importance to human health, with the purpose of mitigating the risks associated with antimicrobial use in food-producing animals. The latest list was published in 2018 [34]. Furthermore, in 2017, the World Health Organization released guidelines on the use of antimicrobials in food-producing animals aiming to preserve the effectiveness of antimicrobials that are important for human health [35].

### Selected developed countries

Here we review national action plans against antimicrobial resistance that were established in the European Union, the United states, Australia, and Japan, leading developed countries in the production and consumption of animal-based products. In addition to national action plans, the following points, regarding actions in the food-producing animal sector, were addressed for each country: (1) Use of growth promotors, (2) Collecting and reporting data on the use of antimicrobials, (3) Operating antimicrobial resistance surveillance systems, (4) Composing a list of critically important antimicrobials for human medicine whose use in food-producing animals should be avoided. These actions are considered necessary to reduce the use of antimicrobials and spread of antimicrobial resistance in food-producing animals.

### Europe

#### Action plans against antimicrobial resistance

In 2011 the European Commission issued an action plan against the rising threats from antimicrobial resistance [36] which was later replaced by the 2017 European Union One Health Action Plan against antimicrobial resistance [37]. The main objectives of this plan, in addition to the objectives of the World Health Organization action plan, included making the European Union a best practice region and boosting research and development in the field.

#### Policy and actions in the food-producing animal sector

*Use of growth promotors.* Some European countries began restricting the use of growth promotors in food-producing animals over two decades ago [38]. Sweden was the first country to ban this practice in 1989 and a total ban on the use of growth promoters was implemented in the European Union in 2006 (Regulation (EC) 1831/2003, [39]).

*Collecting and reporting data on the use of antimicrobials.* In 2009, European Union members began collecting and reporting data regarding the use of antimicrobials in food-producing animals. This step was coordinated and harmonized by The European Medicines Agency through The European Surveillance of Veterinary Antimicrobial Consumption (ESVAC) project. Recently, reporting data on sales and use of antimicrobials in food-producing animals became compulsory [40, 41].

*Antimicrobial resistance surveillance systems.* Surveillance of antimicrobial resistance in bacteria originating from food-producing animals is not coordinated at the European level, although many European countries have such a system in place [42]. The European Union is currently working on assembling the European Antimicrobial Resistance Surveillance network in Veterinary medicine (EARS-Vet), with the aim of reporting on antimicrobial resistance status, following antimicrobial resistance trends and detecting emerging antimicrobial resistance in bacterial pathogens of animals in Europe [43].

*List of critically important antimicrobials to human medicine.* In 2014 The European Medicines Agency published a list of antimicrobials categorized according to their potential to cause increased antimicrobial resistance and danger public health when used in animals [44]. The list was prepared by the Antimicrobial Advice Ad Hoc Expert Group (AMEG) and includes four categories from A-D: Avoid, Restrict, Caution and Prudence. The list was updated in 2020 and The European Medicines Agency advises veterinarians to consider the list when prescribing antimicrobials for animals [44].

Additional important developments have occurred in the animal sector in the European Union since the 2017 action plan and the actions mentioned above, such as the 2020 decision [Decision (EU) 2020/1729] on monitoring and reporting of antimicrobial resistance in zoonotic and commensal bacteria [45] and the adoption of the ‘Farm to Fork’ strategy [46], aimed to accelerate the transition to a fair, healthy and environmentally-friendly food system, while reducing sales of antimicrobials for food-producing animals. In recent years, new regulations on veterinary medicines [Regulation (EU) 2019/6] and medicated feed [Regulation (EU) 2019/4] have entered force and include a ban on prophylactic use of antimicrobials in groups of animals and in medicated feed, restrictions on metaphylactic use of antimicrobials, restrictions on antibiotics reserved for human use and an official obligation to collect and report data on sales and use of antimicrobials in food-producing animals [40, 41].

Implementation of the regulations mentioned above reduced sales of antimicrobials for use in food-producing animals in the European Union by more than 43% between 2011 and 2020 (based on 25 countries, [47]) Moreover, the use of antimicrobials has decreased between 2016 and 2018 and is now lower in food-producing animals than in humans [48]. This trend may have even led to a decline in resistance in indicator *E. coli* in poultry [49].

### The United States

#### National action plans against antimicrobial resistance

In 2013, the United States Centers for Disease Control and Prevention published the first antimicrobial resistance threat report which presented the threats to human health posed by antimicrobial resistance in the United States [20]. A year later, the White House published the United States National Strategy for Combating Antibiotic-Resistant Bacteria [50]. By 2015, the first national action plan, taking a One Health approach, was officially published [51]. The plans five main goals included: (1) slowing the emergence of resistant bacteria and preventing the spread of antimicrobial resistance, (2) strengthening surveillance of resistant bacteria, (3) developing innovative diagnostic tests for identifying resistant bacteria, (4) research and development of new antimicrobials, and (5) improving international collaboration [51]. Since then, the Centers for Disease Control and Prevention has published the second antimicrobial resistance threat report [52] and the White House has released the second national action plan for 2020–2025, which prioritizes infection prevention to slow the spread of antimicrobial resistance and reduce the need for antimicrobial use [53].

#### Policy and actions in the food-producing animal sector

The United States is one of the largest consumers of antimicrobials for animal production [54]. Thus, the government has been implementing different policies to reduce the use of antimicrobials in food-producing animals and combat antimicrobial resistance.

One of the first steps included the Food and Drug Administration 2005 ban on the use of Fluoroquinolones in poultry in order to reduce the prevalence of Fluoroquinolone-resistant *Campylobacter* [55]. This resulted in relatively stable Fluoroquinolones resistant rates in *Campylobacter* over the past years [56]. In 2014 the United States Department of Agriculture published a national action plan summarizing ongoing activities in the field and presenting an integrated plan to enhance the departments efforts to address antimicrobial resistance in food-producing animals[57]. In 2018, the Food and Drug Administration and the Center for Veterinary Medicine published a five-year action plan (2019–2023) to support antimicrobial stewardship in veterinary medicine [58]. Despite these efforts, most of the policies have been implemented voluntarily or incompletely [59].

*Use of growth promotors.* In 2012 and 2013 the Food and Drug Administration published two documents of guidance meant to reduce the use of growth promotors in food-producing animals and encourage therapeutic uses of these antimicrobials, under the supervision of licensed veterinarians [60, 61]. In 2015, the Food and Drug Administration released the Veterinary Feed Directive (VFD), stating that the use of antimicrobials in animal feed requires a veterinary prescription and that antimicrobials should not be used for growth promotion [62]. According to this directive, which was fully implemented in 2017, antimicrobials can be used for disease prevention as long as a licensed veterinarian approved it.

*Collecting and reporting data on the use of antimicrobials.* Currently, the United States government does not collect or report farm-level use of antimicrobials, only data regarding antimicrobial sales by animal species (since 2016; [59]). According to these reports, sales of medically important antimicrobials declined from 2016 to 2020, especially in chickens, yet, rose by 11% between 2017 and 2019 due to an increase in swine production [63].

*Antimicrobial resistance surveillance systems.* Data regarding resistant bacteria from food-producing animals, retail meat products, and foodborne pathogens are collected by the Food and Drug Administration, the Department of Agriculture and the Centers for Disease Control and Prevention as part of the National Antimicrobial Resistance Monitoring System (NARMS). Integrated summary reports are published every few years.

*List of critically important antimicrobials to human medicine.* The Food and Drug Administration published a list of critically important antimicrobials in 2003, which has yet to be revised since [64]. While the World Health Organization’s list refers to all human bacterial diseases, the Food and Drug Administration list focuses only on foodborne pathogens. Other discrepancies between the lists can be found within the ranking system. For example, while the World Health Organization considers fourth‐generation Cephalosporins, Glycopeptides and Polymyxins as “Critically Important”, the Food and Drug Administration categorized them in 2003 as “Highly Important” [64].

### Australia

#### National action plans against antimicrobial resistance

In 1999, the Australian Government established the Joint Expert Technical Advisory Committee on Antibiotic Resistance (JETACAR), which was charged with scientifically examining the threat to human health posed by resistant bacteria arising from agricultural and medical use of antimicrobials [65]. The Joint Expert Technical Advisory Committee on Antibiotic Resistance of Australia published a list of recommendations covering regulation, surveillance, infection prevention, education and research in both human and veterinary medicine, however, most of the recommendations have not been implemented since [65]. In 2013, due to rising global concerns regarding antimicrobial resistance, Australia began forming a national antimicrobial resistance strategy, which was officially published in 2015. An implementation plan for 2015–2019 was published soon after in 2016 [66]. In 2020, the Australian government published its latest national action plan against antimicrobial resistance named ‘2020 and beyond’, in coordination with the World Health Organization’s global One Health action plan on antimicrobial resistance [67].

#### Policy and actions in the food-producing animal sector

Antimicrobial use in food-producing animals in Australia has been restricted to a certain extent since the publication of the UK’s Swann report in 1969 [68]. Key examples include: (1) The ban on Avoparcin in 1997 following the discovery of a link between Vancomycin-resistant enterococcus infections (VRE) in humans and the use of this antibiotic in cattle feed in Denmark [65]. (2) The restriction on registering antimicrobials which might affect human health if used in animals. Accordingly, Fluoroquinolones, Colistin and fourth generation Cephalosporins have never been approved or registered for animal use in Australia, despite their extensive use in agriculture around the world [69]. This action resulted in low levels of Fluoroquinolone resistant *Campylobacter, Salmonella* and *Escherichia* species compared with other countries [70–72].

*Use of growth promotors.* While the Australian government restricts the use of antimicrobials important to human health as growth promotors for food-producing animals [73], antimicrobials which do not match this description are still being used for this purpose, including Avilamycin, Olaquindox and Bambermycin, which are prohibited for use in food-producing animals in the European Union.

*Collecting and reporting data on the use of antimicrobials.* The Australian government does not collect or report farm-level use of antimicrobials and data regarding antimicrobial sales in the country are only partial. The most recent report on antimicrobial sales in Australia was published in 2014 by the Australian Pesticides and Veterinary Medicines Authority (APVMA). This report showed no significant change in the total quantity of antimicrobial products sold from 2005 to 2010 [74].

Antimicrobial resistance *surveillance systems.* Australia does not currently have a national surveillance system for monitoring antimicrobial resistance in bacteria derived from food-producing animals.

*List of critically important antimicrobials to human medicine.* Australia published a list of critically important antimicrobials in 2018 [75]. This list differs from the World Health Organization list due to different antimicrobial treatment practices in human and animal medicine. One noticeable difference is that the Australian list, unlike other country lists, classifies Macrolides as “Low Importance” to human and veterinary medicine [75].

### Japan

#### National action plans against antimicrobial resistance

The Japanese government published its first national action plan against antimicrobial resistance in 2016 [76], as part of the global effort to combat antimicrobial resistance. Like other countries, this plan took a One Health approach and was based on the World Health Organization’s Global Action Plan published a year before. To implement the national action plan, the government established the Antimicrobial Resistance Clinical Reference Center (AMRCRC) and the Antimicrobial Resistance Research Center, which integrate surveillance data on humans, animals and the environment and perform awareness-raising activities in Japan [76, 77]. An updated national action plan was expected to be released in 2021, yet due to the emergence of the 2019 Coronavirus disease (COVID-19), this has been delayed [78].

#### Policy and actions in the food-producing animal sector

*Use of growth promotors.* Japan only partially restricted the use of growth promoters in feed of food-producing animal due to European Union reforms on the matter [1]. As of 2018, the use of Colistin as a food additive in animal feed has been prohibited by the Food Safety Commission of Japan [79].

*Collecting and reporting data on the use of antimicrobials and antimicrobial resistance surveillance.* The Japanese Veterinary Antimicrobial Resistance Monitoring System (JVARM) was established in 1999 following the entry into force of the law on ‘food, agriculture and rural areas’ (aimed to ensure food safety and improve food quality) and due to the global concern regarding the impact of antimicrobial resistance on human health [80]. The main objectives of The Japanese Veterinary Antimicrobial Resistance Monitoring System were to monitor antimicrobial resistance in bacteria from food-producing animals and to monitor the consumption of antimicrobials in food-producing animals. The system's latest report (2020) for the years 2016–2017, showed a general decrease in antimicrobial sales intended for food-producing animals between 2001 and 2014, however, an opposite trend was observed in 2015–2017 [80].

*List of critically important antimicrobials to human medicine.* Japan published a list of critically important antimicrobials in 2014 [81].

### Selected Middle Eastern countries

Here we review national action plans against antimicrobial resistance that were developed in Egypt, Jordan and Lebanon, Middle Eastern countries neighboring Israel. According to the 2021 World Health Organization tripartite antimicrobial resistance country self-assessment survey [82] and based on country responses to this survey, Egypt, Jordan and Lebanon have developed national action plans against antimicrobial resistance. Moreover, Egypt and Jordan have begun implementing their national action plans.

- *Egypt.* The Egyptian government began drafting its national action plan in 2015 based on the World Health Organization’s Global Action Plan, with a special emphasize on One Health. The four-year national action plan (2018–2022), coordinated by the Ministry of Health and Population (MOHP) with the support of World Health Organization, was published in 2018 [83].
- *Jordan.* Like Egypt, Jordan released its four-year national action plan (2018–2022) against antimicrobial resistance with the support of World Health Organization’s in 2018. This national action plan was also based on the World Health Organization’s Global Action Plan, emphasizing the One Health approach [84].
- *Lebanon.* Lebanon published its national action plan against antimicrobial resistance in 2019, in accordance with the World Health Organization’s Global Action Plan [85].

According to a recent review assessing the alignment of different national action plans with the World Health Organization’s Global Action Plan on antimicrobial resistance [86], Egypt, Jordan and Lebanon fully addressed objective two and four of their national action plans (improving knowledge through surveillance and optimizing the use of antimicrobial agents, respectively). Objective five (ensuring sustainable investment in countering antimicrobial resistance) was partially addressed by Egypt. All three countries currently lack financial resources for antimicrobial resistance activities [86].

### Combating antimicrobial resistance in Israel

Although the Israeli government has recently developed a national action plan against antimicrobial resistance based on the One Health approach, it has yet to publish or implement it. While a few steps aimed to reduce the spread of antimicrobial resistance in food-producing animals have been taken over the past years (detailed below), notable loopholes in the Israeli veterinary drug regulatory system have, and are still likely to, limit the success of these actions. This section will address the use of growth promotors, collection of data on the use of antimicrobials, antimicrobial resistance surveillance systems and the status of a list of critically important antimicrobials to human health in Israel.

#### Overview of the Israeli veterinary drug regulatory system

Drugs in Israel are required to be registered in order to be marketed and used. Veterinary drugs in Israel are evaluated and registered by the Pharmacy Department in the Ministry of Health, in coordination with the Veterinary Services and Animal Health unit in the Ministry of Agriculture and Rural Development. According to the Israeli Drug Registry [87], as of 2020, 349 (7%) of the currently registered drugs (~ 5,000) are veterinary medicines, of which 215 (61%) are intended for food-producing animals, mainly poultry (Fig. 1a). Over half (59%) of these drugs contain antimicrobials (Fig. 1b), mostly Sulfonamides, Fluoroquinolones and Macrolides (Fig. 1c). In recent years, Israel has harmonized the process of veterinary drug registration with European Union regulations.

The Israeli legislation allows for importation of non-registered drugs under article 29 C of the Pharmacist Regulations—Medicinal Products [88], as long as: (1) there is no alternative drug registered in Israel, (2) the drug is imported by wholesalers from an authorized country in which it is already registered, (3) the drug is prescribed by a licensed doctor/veterinary. This legislation is meant to be a quick and effective way of obtaining drug supply in shortage [89]. Article 29 C is widely used to import non-registered veterinary drugs to Israel, including antibiotics which were used in the past as growth promotors. Between 2017 and 2019, 380 unregistered veterinary drugs were imported into Israel under article 29 C, a third of them intended for food-producing animals (Table 2).

**Fig. 2:**
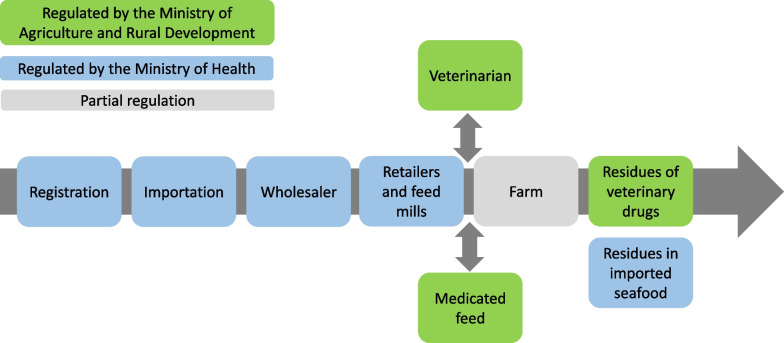
The supply chain of drugs for food-producing animals in Israel and the division of government supervision upon it, as of 2022. The Ministry of Health is responsible for regulating registration and importation of veterinary drugs, as well as supervising wholesalers, retailers and feed mills. The Ministry of Agriculture and Rural Development is responsible for regulating veterinary best practices, medicated feed and for monitoring residues of veterinary drugs in products of animal origin. The responsibility for monitoring residues of veterinary drugs in seafood belongs to the Ministry of Health. Currently, farms are only partially regulated by the Ministry of Agriculture and Rural Development

Veterinary drugs are imported by wholesalers and distributed to retailers. Wholesalers are regulated by the Good Manufacturing Practice (GMP) unit in the Ministry of Health of Israel according to the European Union good distribution practice (GDP) standards (Fig. 2). Drugs for food-producing animals are supplied to farmers through the retailers and feed mills by written prescription only, which should be documented and filed per sale. Retailers and feed mills do not require the presence of a pharmacist, rather they need to hold a “toxins license” issued by the Ministry of Health of Israel (renewed annually), which lists drugs kept in storage. The “toxins license” includes registered drugs only, yet retailers and feed mills also supply farmers with non-registered drugs (*i.e.,* drugs imported under article 29 C). In the past both the Veterinary Services and Animal Health unit and the Ministry of Health inspected the retailers and feed mills, however in 2016, a change was made to the Israeli Pharmacist Ordinance [90] authorizing only Ministry of Health personal to inspect these facilities (Fig. 2). Consequently, the Veterinary Services and Animal Health unit lost their authority to inspect retailers and feed mills, despite being present in these facilities on a regular basis. This resulted in reduced inspection capacity on this important link in the chain of supply of veterinary drugs. Furthermore, inspection methods by the Ministry of Health personal currently differ between districts.

It is important to note that the Veterinary Services and Animal Health unit constantly monitors residues of veterinary drugs (including antimicrobials) in products of animal origin (meat, eggs, etc.; Fig. 2), publishing the results in annual reports. These reports mostly demonstrate low residues in all product types [91], although relatively high residues have been observed for Tetracyclines (the antibiotic used most in food-producing animals in Israel), especially in 2016 and 2018 [92].

#### Actions taken to combat antimicrobial resistance in food-producing animals in Israel

As done previously, we will address the use of growth promotors, collection of data on the use of antimicrobials, antimicrobial resistance surveillance systems and the status of a list of critically important antimicrobials in Israel.

In 2012, a joint committee including the Veterinary Services and Animal Health unit and the National Center for Infection Control in the Ministry of Health was established with the purpose of coordinating actions against the spread of antimicrobial resistance in food-producing animals, as done by numerous countries during this time period. Throughout the past decade the committee’s activity included conferences on antimicrobial resistance. In 2019, the committee established a team of professionals from the Ministry of Agriculture and Rural Development and the Ministry of Health meant to prepare an outline for a national action plan against antimicrobial resistance. While the action plan has been drafted since (2022), it has yet to be published. In 2020, a comprehensive document proposing steps to be taken in order to develop and implement a One Health national action plan in Israel was published by the Ministry of Health [93]. Recently the Ministry of Agriculture and Rural Development performed a regulatory impact assessment (RIA) regarding prudent use of antimicrobials in food-producing animals. This assessment concluded that farmers and veterinarians should report on the use of antimicrobials via a digital system. A committee, meant to accompany the establishment of the system, is presently being organized.

*Use of growth promotors.* A total ban on the use of growth promoters entered into force in 2018, following the 2014 legislation regarding animal feed [94]. Prior to this legislation, antimicrobials used for growth promotion were registered in Israel as feed additives and did not require a veterinary prescription to be used. The new law changed the status of these antimicrobials from feed additives which could be purchased ‘over the counter’ to medication requiring a veterinary prescription. Furthermore, it prohibited metaphylactic use of antimicrobials in food-producing animals. Since then, only two antimicrobials previously used for growth promotion have been officially registered in Israel—Avilamycin and Bacitracin. Currently, article 29 C is used to import non-registered growth promotors. Nonetheless, these antibiotics are not to be used for metaphylaxis as per the 2014 legislation.

*Collecting and reporting data on the use of antimicrobials.* The Israeli government does not currently collect or report farm-level use of antimicrobials or data regarding antimicrobial sales. The main barrier to date being the demand of wholesalers to protect confidential commercial information. In 2014, the Veterinary Services and Animal Health unit performed a survey in which sales data were collected from wholesalers on a voluntary basis. The results (standardized by population correction unit—PCU and by the food-producing animal composition) indicated that the total use of antimicrobials in food-producing animals in Israel was four times higher than the average use in selected European countries [29]. A similar trend was demonstrated in a follow-up survey conducted in 2018 [92].

*Antimicrobial resistance surveillance systems.* While surveillance of antimicrobial resistance in humans is well developed, there is no centralized surveillance system for antimicrobial resistance in food-producing animals in Israel. Currently data regarding resistant bacteria from foodborne pathogens (specifically *E. coli O157:H7 serotype, Salmonella spp. and Campylobacter spp.*) are collected by the Government Central Laboratories in the Ministry of Health, which publish their findings in annual reports. According to the latest report for 2020, all monitored foodborne pathogens demonstrate high multi-drug resistance rates [30]. The Kimron Veterinary Institute (diagnostic and research arm of the Veterinary Services and Animal Health unit), the Egg and Poultry Board and the Israeli Dairy Board test antimicrobial resistance in bacterial pathogens of animals for the purpose of clinical treatment. These pathogens are not monitored regularly, rather they are tested based on the occurrence of infectious diseases or upon specific requests from farmers. A plan to monitor antimicrobial resistance in slaughterhouses has been suggested recently by the Veterinary Services and Animal Health unit—Ministry of Health joint committee, but it has not been implemented yet.

*List of critically important antimicrobials to human medicine.* While Israel does not have a list of its own, the Veterinary Services and Animal Health unit recently adopted the European Union recommendation (Regulation (EU) 2017/625, [95]) prohibiting the use of substances from category A of The European Medicines Agency’s list of critically important antimicrobials. Following this decision, the Ministry of Health prohibited importing category A substances to Israel. Currently, as part of the regulatory impact assessment regarding prudent use of antimicrobials in food-producing animals, it has been suggested that the Israeli government fully adopt the European Unions list of critically important antimicrobials.

## Discussion

While Israel has made a few policy changes regarding the use of antimicrobials in humans and animals in recent years, it is far behind in the implementation of a One Health national action plan compared to other developed countries. To date, growth promotors have been banned and a partial antimicrobial resistance surveillance system of foodborne pathogens exists, however, collecting and reporting data on the use of antimicrobials, a highly necessary step to evaluate the current state in Israel, is still lacking. This is likely one of the main reasons for the delay in implementing a national action plan against antimicrobial resistance in Israel. Another likely reason would be the separation of powers and partial cooperation between the Ministry of Health and the Ministry of Agriculture and Rural Development, as described above. Unless amended, significant loopholes in the Israeli legislation of veterinary drugs are likely to further delay the implementation of a national action plan and limit the success in affecting antimicrobial use reductions. Several actions can be performed in the food-producing animal sector to promote the fight against antimicrobial resistance in Israel:

*Awareness.* One of the main objectives of the World Health Organization’s global action plan against antimicrobial resistance is to improve public awareness and understanding of antimicrobial resistance [32]. Currently, there is a lack of public awareness in Israel regarding the health and environmental impacts of uncontrolled use of antimicrobials in humans and animals. It is therefore essential to enhance awareness among the general public, but also amongst target groups, including farmers and health practitioners from both human and animal sectors. This can be done, for example, by creating a web-page dedicated to explaining antimicrobial resistance in the Ministry of Health and the Ministry of Agriculture and Rural Development websites, participating in the World Antimicrobial Awareness Week [96], conducting surveys to understand the level of awareness and spreading knowledge through campaigns, social media and networks (as successfully done in Israel for COVID-19). Professional conferences and courses could be used to improve awareness among farmers and health practitioners in all sectors.

*Data collection on farm-level antimicrobial use.* Data on antibiotic use in food-producing animals are necessary to understand the public health risk associated with its use. This could be done in Israel using an online prescription system, such as the Danish government's VetStat system [97]. VetStat is shared by pharmacies, veterinarians, farmers and feed mills, and it enables registration of antimicrobial use at the herd level on a monthly basis, based on medical prescriptions. Of note, a similar database for humans exists in Israel—health maintenance organizations register all prescriptions to patients online. Furthermore, the National Center for Infection Control in the Ministry of Health monitors the use of antimicrobials in the human health sector, including hospitals, post-acute care hospitals, and the community (*i.e.,* “Kupot Holim”). To date, this step has been delayed in the animal sector due to the demand of wholesalers to protect confidential commercial information. With the right legislation, data from platforms of animal and human health sectors could be used to monitor the prescription of antimicrobials and the development of resistance in bacteria in both animals and humans. Collecting data regarding the use of antimicrobials in humans and animals is relevant to both the Ministry of Health and the Ministry of Agriculture and Rural Development and cooperation between the two is essential (and a possible barrier) to promote this important step. Such an online drug prescription system could also prevent sales of antimicrobials without prescriptions or with a retroactive prescription (*i.e.,* prescriptions provided to the retailer by the veterinarian only after antimicrobials were already sold to the farmer).

*Centralized antimicrobial resistance surveillance system.* There is no centralized surveillance system for antimicrobial resistance in food-producing animals and humans in Israel. Furthermore, antimicrobial resistance is not monitored equally across all sectors. For example, while antimicrobial resistance in humans and foodborne pathogens are strictly examined, resistant bacteria in slaughterhouses are not yet monitored and antimicrobial resistance of infectious diseases of animals are monitored partially. There is an urgent need to set up a formal database which integrates data on antimicrobial resistance from all sectors (human, animal and environment). Surveillance techniques should be standardized among laboratories and the data harmonized among the sectors to allow comprehensive monitoring of drug-resistant bacteria and to provide a basis for taking action to control antimicrobial resistance.

*Mitigation through farm biosecurity.* Biosecurity refers to hygienic practices meant to prevent the introduction or spread of infectious diseases, such as limiting animal and human movement between farms, avoiding mixing animal breeds, reducing fecal contamination and reducing overcrowding. Implementing hygienic and biosecurity protocols in farms in Israel is a plausible way of preventing disease and thus the need for antimicrobials for treatment. These protocols may also reduce the need for preventive use of antimicrobials. Indeed, biosecurity has been shown to reduce the need for antimicrobials in food-producing animals (*i.e*., [98–100]) and it has even already been successfully applied in some poultry farms in Israel. Transitioning farmers to biosecurity protocols will require incentive and funding from the government (a likely barrier of this step), as they are likely to increase the economic burden on farmers, and consequently increase the price of food. Providing government funding will reduce the economic burden on farmers and may mitigate resistance on their behalf.

*Antimicrobial stewardship in food-producing animals.* Attempts to reduce the use of antimicrobials in food-producing animals, such as prohibiting the use of growth-promoters or collecting data on the usage of antimicrobials, has led to reductions in antibiotic resistance in food-producing animals worldwide. However, this alone it not enough and further measures are needed to enhance antimicrobial stewardship at the farm-level. These measures should include disease prevention (via farm biosecurity, see above), using an evidence-based approach to diagnose diseases and determine whether there is a need to use antimicrobials (*i.e.,* indication for antimicrobial use), and using antimicrobials judiciously—appropriate dosage and duration of antimicrobials, and assessing the outcome of their use. An initial step towards implementing these measures should be developing a stewardship plan, as seen in the European Union [101].

*List of critically important antimicrobials to human medicine.* Israel does not have a list of its own, rather it partially adopted the European Unions list of critically important antimicrobials [95]. The World Health Organization recommends each country develop a national list of critically important antimicrobials based on human health practices in the specific area [34]. Since Israel exports many of its animal-based products to the European Union, it seems adequate to fully adopt the European Union list, while considering the regional requirements in human medicine, as suggested in the regulatory impact assessment, conducted by the Ministry of Agriculture and Rural Development.

*Legislation and regulation.* There is a need for laws and/or regulations in Israel regarding antimicrobial resistance in several aspects: (1) There is no regulation regarding the collection of data on antimicrobial use from wholesalers, retailers or farmers. There is a need to add an article to the Israeli Pharmacist Ordinance [90] requiring first wholesalers, and further on retailers or the farmers, to collect and report data on sales and use of antimicrobials in food-producing animals, similar to the European Union. This requirement should be part of the registration process of new veterinary drugs. (2) There is a need to amend the 2016 change to the Israeli Pharmacist Ordinance [90] preventing the Veterinary Services and Animal Health unit personal from inspecting retailers and food mills which provide farmers with veterinary drugs. This will increase the inspection capacity and enhance cooperation between the Ministry of Health and the Ministry of Agriculture and Rural Development. (3) There is a need to tighten regulation on importation of non-registered drugs under article 29 C by establishing an online system shared by the Ministry of Health and the Ministry of Agriculture and Rural Development for managing and documenting all stages of this process. Only drugs which are currently undergoing the registration process should be allowed to be imported via article 29 C. There should be a ban on the importation of antimicrobials previously used as growth promotors. Finally, enhancing the cooperation between the Ministry of Health and the Ministry of Agriculture and Rural Development will ensure non-registered drugs are imported for clinical need only. This could be done by amending the 2016 change to the Israeli Pharmacist Ordinance (see above) and by enhancing the activity of the ministries’ joint committee for coordinating actions against the spread of antimicrobial resistance, to include not only conferences on antimicrobial resistance, but also activities such as reviewing antimicrobial resistance and drug use data in food-producing animals.

Sustaining a national network to monitor and control antimicrobial resistance is one of the greatest barriers as it requires funding and the support of policymakers. Currently, the implementation costs of a national action plan against antimicrobial resistance in Israel remain unknown, from the government level down to the farm level, including the effects on product supply and the price of food. A cost/benefit analysis should be conducted, considering the financial costs involved in the misuse of antibiotics in both human and animal sectors, including the impact on the environment. Currently there is no funding source for antimicrobial resistance activities in Israel, such as establishing an online drug registration system and a centralized antimicrobial resistance surveillance system, transition of farmers to facilities with high biosecurity, antimicrobial resistance research in Israel and awareness campaigns. A first step towards this could include using the World Health Organization’s costing and budgeting tool meant to support countries in budgeting antimicrobial resistance activities [102].

## Conclusions

Antimicrobial resistance poses a major threat to human and animal health worldwide and it is considered as one of the principal public health threats of the twenty-first century. In recent years, many countries began developing and implementing national action plans against antimicrobial resistance. Although some achievements can be counted, the Israeli government has yet to implement a One Health based national action plan against antimicrobial resistance. This national action plan should address the following:

- Collecting and reporting data on the use of antimicrobials in humans and animals using an online system (as suggested in the regulatory impact assessment of the Ministry of Agriculture and Rural Development)
- Operating a centralized surveillance system for antimicrobial resistance in humans, animals and the environment
- Improving the awareness and understanding of antimicrobial resistance in the general public and in health practitioners from human and animal sectors
- Addpoting the European Union list of critically important antimicrobials
- Enforcing and supervising best practices of antimicrobial use at the farm-level
- Reducing incidence of infection through farm biosecurity
- Supporting research and development of new antimicrobial treatments, vaccines and diagnostic tools

Recent studies show that like other countries in the world, antimicrobial resistance is also widespread among food-producing animals in Israel and the use of antimicrobials in farms is high. Without a comprehensive and funded national action plan, the risks of antimicrobial resistance to the public health in Israel will continue to increase and Israel will remain behind in the global effort to fight antimicrobial resistance.

## Data Availability

Not applicable.
